# Analysis of Energy and Material Consumption for the Manufacturing of an Aeronautical Tooling: An Experimental Comparison between Pure Machining and Big Area Additive Manufacturing

**DOI:** 10.3390/ma17133066

**Published:** 2024-06-21

**Authors:** Alejandro Marqués, Jose Antonio Dieste, Iván Monzón, Alberto Laguía, Carlos Javierre, Daniel Elduque

**Affiliations:** 1 R&D Department, Fundación Aitiip, Polígono Industrial Empresarium, C/Romero, No. 12, 50720 Zaragoza, Spain; joseantonio.dieste@aitiip.com (J.A.D.); ivan.monzon@aitiip.com (I.M.); alberto.laguia@aitiip.com (A.L.); 2 Department of Mechanical Engineering, University of Zaragoza, C/María de Luna, 50720 Zaragoza, Spain; carlos.javierre@unizar.es (C.J.); delduque@unizar.es (D.E.)

**Keywords:** additive manufacturing, big area additive manufacturing, energy consumption, material consumption, wire arc additive manufacturing, aeronautical tooling

## Abstract

Additive manufacturing (AM) has been fully incorporated into both the academic and the industrial world. This technology has been shown to lower costs and environmental impacts. Moreover, AM-based technologies, such as wire arc additive manufacturing (WAAM), have been proven suitable for the manufacturing of large products with significant mechanical requirements. This study examines the manufacture of two aeronautical toolings: first, using conventional techniques, and second, using a big area additive manufacturing (BAAM) process, specifically WAAM technology, followed by second-stage hybrid machining. Both toolings can be considered interchangeable in terms of design and performance. Energy and material consumption were analysed and compared throughout both tooling procedures. The results show the important optimisation of both procedures in manufacturing WAAM tooling, encompassing the additive process and second-stage hybrid machining. Nevertheless, the time required for WAAM tooling manufacturing increased significantly compared to conventional manufacturing tooling. Moreover, based on metrology data from the AM process, a theoretical study was conducted to assess different design optimisations for WAAM tooling manufacturing and determine their influence on material and energy consumption. These theoretical results improve those already obtained regarding energy and raw material savings.

## 1. Introduction

Due to the material and energy crisis [1,2] and following the environmental awareness trend of the last few years [3,4,5,6,7], additive manufacturing (AM) has been gaining relevance in the industrial environment. In this vein, the *Annual Additive Manufacturing Trend Report* produced by HUBS, which looks at the international trends and uses for AM based on responses from 1504 engineering businesses, shows a significant increase in the use of AM technologies in the past year. It also highlights the increased media exposure of these technologies since the COVID-19 pandemic, as the ability to create PPE (Personal Protective Equipment) rapidly made the technology very popular [8]. Moreover, this technology has already proven its ability to be fully implemented in the industry [9,10,11], being used to manufacture functional metallic parts and entering an increasing number of industrial fields, such as nuclear [12,13], construction [14,15], railway [16], and military applications [17]. Its capacity to create complex geometries and maintain good mechanical properties [18,19,20,21] while potentially saving materials and energy compared to conventional manufacturing strategies [22,23,24] has made it a widely researched technology. In engineering, we highlight the procedure known as BAAM (big area additive manufacturing), which is nothing more than a dimensional specification of the AM process. The term BAAM encompasses additive manufacturing machines capable of working in larger dimensions than those limited by standard 3D printers, being able to reach printed volumes of several cubic metres [25,26,27,28,29].

As reflected in the previous paragraph, AM is a widely used manufacturing process in many industries and, in addition to the characteristics mentioned above, another of its benefits is the ability to work with various types of materials. The different printing processes encompassed by AM allow for the use of not only metallic materials [30,31,32] but also more complex materials, such as composites [33,34,35,36], recycled materials [37,38], and biological materials [39,40]. This article focuses on metal additive manufacturing (MAM) since its ultimate goal, as explained later, is manufacturing a functional core mould.

In order to introduce metallic additive manufacturing (MAM) technologies, two groups stand out from the rest, depending on their feeding material: powder-feed technologies and wire-feed technologies. Within these groups, the most representative AM technologies are Laser Metal Deposition (LMD) and Powder Bed Fusion (PBF) when referring to powder-feed technologies, and wire arc additive manufacturing (WAAM) when referring to wire-feed technologies.

According to ISO/ASTM 52,900:2021 [41], Powder Bed Fusion is a standard process, in which thermal energy selectively fuses regions of a powder bed [42]. This AM technology creates extremely complex geometries with a high surface finish and small details. Its main limitation is the reduced size of its printed area, limited by the printer size (400 × 400 mm in general). On the other hand, WAAM is a fusion manufacturing process, in which the heat energy of an electric arc is employed for melting the electrodes and depositing material layers for wall formation or for the simultaneous cladding of two materials to form a composite structure [43,44]. This technology creates large final geometries with low surface finish quality, demanding hybrid machining on surfaces with high geometrical demands [45]. Moreover, it is a constantly evolving technology, whose future applications in the field of metal part repair are being extensively researched [46].

Several studies have developed modelling methods comparing manufacturing processes with pure machining, studying the optimal strategy for specific part manufacturing. Several authors [47,48,49] introduced a decision-making model to calculate the suitability of choosing a PBF procedure versus a pure machining one. The study conducted by Campatelli et al. [50] specifically compares the WAAM-subtractive process (WAAM manufacturing with a manufactured finishing process) with a pure subtractive manufacturing process for the same final geometry, a blade designed according to the NACA 9403 standard [51], with a 100 mm chordal length and made of EN S235JR structural steel.

In this case, the WAAM method is Gas Metal Arc Welding (GMAW)-based additive manufacturing [52]. In the GMAW procedures, the heat of the arc generated between the consumable electrode and the workpiece is used to melt the surfaces of the base metal and the tip of the electrode. The molten metal from the electrode is transferred to the workpiece through the arc, where the substrate is deposited. The welding torch provides the weld with an inert-gas-protected atmosphere, wire input, and welding current [53]. This technology’s main advantages are a higher deposition rate, lower equipment cost, and fewer residue issues. However, a primary concern in this technique is the deterioration of the surface quality on the side face of the fabricated parts, making it necessary to post-process surfaces that require precision finishing [54,55,56].

Three criteria were applied to select the most suitable MAM technology for study: the accomplishment of BAAM conditions, energy consumption, and material consumption. As mentioned before, Powder Bed Fusion technology is constrained by its printing area, limiting the possibilities of this technology. Furthermore, its low feeding rate makes this technology the least optimal to achieve BAAM constructions [57]. Within the second selected criteria, energy consumption was considered regardless of its final geometry complexity [58]. Several studies conclude that Powder Bed Fusion technologies require an energy demand higher than one order of magnitude to accomplish the same printed geometry as WAAM technology [59].

**Figure 1:**
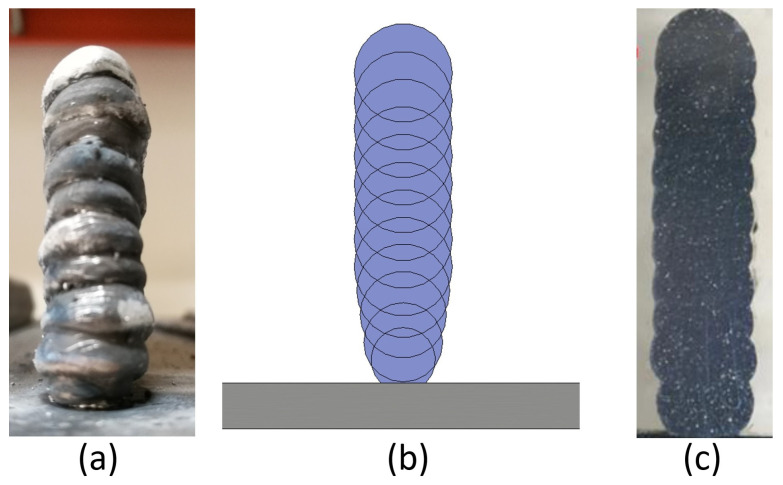
Overlapping weld bead structure in the WAAM process. (**a**) A photograph of the structure. (**b**) A geometric representation of the distribution of weld seams. (**c**) A photograph of a cross-section of the structure.

The WAAM manufacturing process is based on the superposition of layers. Due to this feature, the lateral surfaces of these constructions show a geometry similar to the one displayed in Figure 1. For this reason, the WAAM process is considered a Near to Net Shape technology. Once the process has ended, the result obtained is not the final part but a rough previous workpiece that an NC machine should finish. Settled WAAM building parameters affect the final surface quality [60,61,62]; nevertheless, the study by Lopes et al. [63] shows that the mechanical behaviour of the as-built components does not significantly influence the milling process.

This uneven lateral surface results from a compromise reached by optimising the parameters of the welding temperature (dependent on electrical current) and printing speed [64]. These parameters affect the height of the weld bead and its width, which are inversely proportional.

For large constructions, a narrow weld bead is desirable, allowing high bead growth, even though it reduces the weld penetration into the bottom layer and creates the aforementioned rounded ball-shaped bead [65]. Moreover, these parameters affect the material’s final properties, so their range of modification is modified based on the experience accumulated with this technology and the existing literature [66,67,68,69,70].

This study aims to compare a pure subtractive manufacturing process versus a whole BAAM process in the manufacturing of aeronautical tooling, focusing on energy and material consumption. For this purpose, two twin toolings were constructed, one manufactured by pure machining and the other one manufactured by a combination of WAAM technology and a second-stage machining process. Throughout both processes, energetic inputs have been controlled, both in the AM process and pure milling, as well as the total material consumed. Additionally, in this study, different WAAM design strategies have been subjected to a sensitivity analysis, comparing the different theoretical outputs obtained with those used on the real printed WAAM tooling.

## 2. Materials and Methods

As introduced, this article compares the energetic and material outputs obtained from two similar toolings produced from two different manufacturing methods. One was produced by conventional manufacturing methods, i.e., starting from a block of raw material and carrying out milling processes on a numerical control (NC) machine until the final geometry was obtained. On the other hand, the second tooling was produced from scratch by an AM method, and subsequently underwent a series of milling processes in an NC machine until reaching the desired final geometry. Due to the data provided by researchers in the previous paragraphs, the WAAM process was chosen as the most optimal AM process for the selected application. Owing to the size of the final tooling (500 × 450 × 150 mm approximately), the additive manufacturing procedure is considered a BAAM process.

As mentioned in the previous section, the additive manufacturing process characteristics condition both the part’s design process procedures and its subsequent construction. One of the most notable restrictions is the mentioned rounded ball-shaped bead. Its irregular geometry, which extends in all areas perpendicular to the printing direction, forces a specific printing Computer-Aided Design (CAD) architecture for the AM parts, in which an over-thickness is implemented on the outer surfaces. This specific CAD has been named the WAAM-process-adapted CAD. Moreover, to avoid problems related to small failures in the printing procedure [71,72,73], such as a lack of material or occasional welding errors, every surface was designed with this over-thickness during the WAAM-process-adapted CAD. This over-thickness was initially set at 10 mm. To achieve this theoretical 10 mm over-thickness, previous printing tests in the same environment were reviewed. All parameters, such as the printing strategy, weld control software, and printing materials (filler material and inert gas), were previously tested, although always on smaller constructions. These tests concluded that a safety over-thickness of 5 mm was sufficient to ensure the construction of the original geometry in small parts. However, as this construction was much larger than those carried out for the tests, and considering the investment in time and material, it was decided to increase the theoretical over-thickness. This way, the chances of a specific welding error resulting in an area lacking material were reduced, although this meant an increase in the material consumed and the printing time.

In contrast with other AM technologies, the WAAM process is performed without any extra supporting structure. Consequently, support-free constructions performed by this printing method may suffer from errors of precision or even material detachment in areas where the cantilever angles are too high [74,75]. Liu et al. [76] showed that thin walls of construction with a theoretical 25° inclination showed an error of up to 6%. As in the case of the design over-thickness, the WAAM-process-adapted CAD was designed conservatively considering, in addition to the abovementioned article, the experience gained from previous tests. Therefore, the maximum design cantilever angle was set at 20°.

Focusing on the tools to be studied, the first tooling was manufactured in a common NC machine with a pure machining method. To clearly differentiate the machining procedures applied to each tooling, this traditional machining method was named pure machining. At the same time, the twin tooling mentioned above was an AM technology-made tooling, finished by a second-stage hybrid-machining process, referred to as hybrid mechanisation in this article. The main differences in both toolings were the design specifications, in order for them to work with the respective procedure restrictions but be exchangeable during the final complete aeronautical tooling.

**Figure 2:**
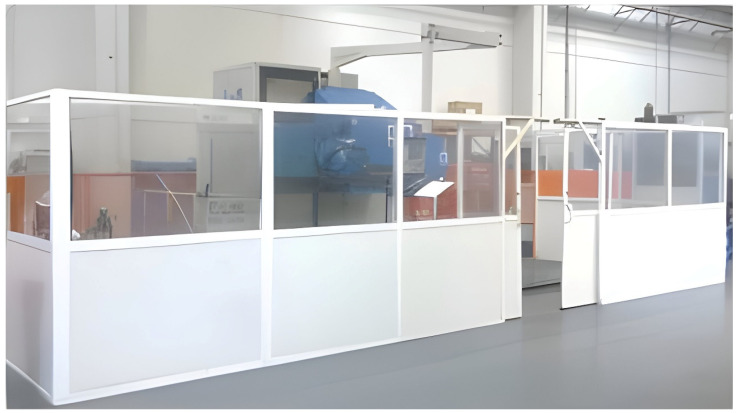
The 5-Axis NC machine, CME FCM 400. Photograph taken at the facilities of Fundación Aitiip.

Referring to machining procedures, both pure and second-stage WAAM technology hybrid-machining processes were performed using a 5-Axis NC machine. The machining process executed was a milling process, and the chosen machining centre was a CME FCM 400 (Figure 2). The energetic input information for this procedure was characterised according to the measured inputs obtained in real-time.

The NC machine electric consumption, throughout both toolings’ machining processes, was recorded by a Circutor C-80 system analyser [77], which displays and stores power consumption data. It should be noted that this study included a gate-to-gate analysis regarding the additive and subtractive manufacturing processes. Therefore, the energy needed to manufacture the raw block, welding wire, and shielding gas was not considered.

**Figure 3:**
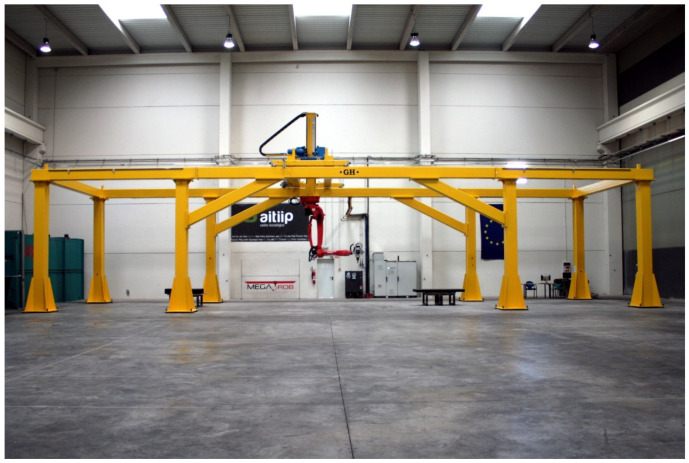
KRAKEN—Hybrid manufacturing robotic system. Photograph taken at the facilities of Fundación Aitiip.

On the other hand, regarding the WAAM tooling, an AM system designed to fabricate large parts was used. This system was developed in the KRAKEN project within the framework of the European Union’s Horizon 2020 Research and Innovation Program under grant agreement No. 723,759 [78]. The Kraken system is based on a robotic arm (COMAU NJ130) [79] mounted on a gantry crane. This distribution removes the dimensional restrictions on the gantry crane structure, so the working space is not limited by the machine itself but to the assembly space available within the gantry crane structure. Figure 3 displays an overview of the Kraken system. The mentioned robotic arm counts on interchangeable heads and can perform milling, polymer resin extrusion, and additive manufacturing processes based on WAAM technology. The combination of crane mobility with the WAAM process head enables the manufacturing of BAAM parts.

Regarding software, robot movements are programmed directly from CAM software (HyperMILL CAD/CAM 2023) [80] to automatically guide the robot’s path. The movements obtained are post-processed for ISO G-Code language. The file obtained is simulated in an Off-line Programming Software (Ultimaker Cura 2023) [81] to avoid collisions and singularities and ensure that all the movements are within the reach scope of the robot. The power source platform utilised in this procedure was a Fronius TPS 400i [82].

The robotic arm position was recorded in real-time. At the same time, the construction height was measured by a laser distance sensor (Keyence CMOS Multi-Function Analogue Laser Sensor) [83] installed in the welding torch, and the temperature was controlled by a temperature sensor (OPTRIS—3MH CF) [84]. These height and temperature sensors created two independent security loops:

1. A distance control system at the WAAM torch was programmed to control the ‘layers’ height. This system measures the distance between the torch and the last welded layer, comparing that input data with the theoretical distance of the printing simulation. If the measured data are within a predetermined scale, the procedure continues. Otherwise, the program skips a printing layer if the distance measured is smaller than the theoretical distance or repeats it if it is higher.
2. The temperature sensor creates a second security loop, in which the last printing layer temperature is measured. This sensor is installed in the welding head, 25 mm behind the welding torch, measuring the temperature of the weld bead immediately after its deposition. High layer temperatures lead to geometrical inconsistencies in the geometry, and if too low, lead to interlayer-adhesion problems. This second low-temperature issue is less common and problematic. Thus, a minimum time between layers was used to ensure a layer temperature lower than 250 °C, even if geometrical singularities distort the temperature signal.

Data collection for the WAAM process was carried out based on the main welding equipment (Fronius TPS 400i), which measures the material supplied, whose parameter is defined as the WFR (Wire Feed Rate), electric current, and the voltage of the system, transferring them to a database via a Profibus communication.

Finally, based on the results obtained for the already-printed WAAM tooling, a sensitivity analysis was performed to test the over-thickness parameter selected. An assimilation with different design over-thicknesses for WAAM-process-adapted CAD was performed. These theoretical toolings were correlated with the real toolings, employing their designed volume parameters. By performing this assimilation, outputs comparable to the final test results were obtained theoretically.

The final aim of these theoretical models is to calculate the minimum over-thickness parameter designed for the WAAM-process-adapted CAD to minimise both material and energy consumption. This parameter should also achieve geometrical restrictions and not compromise the correct functioning of the tooling during the process.

### 2.1. Case Study

**Figure 4:**
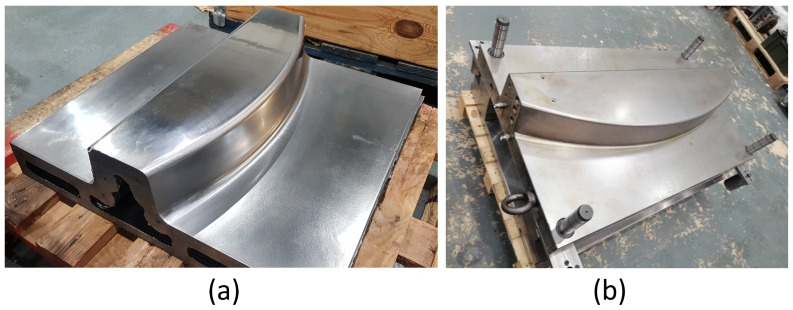
Final tooling comparison between (**a**) WAAM tooling and (**b**) pure machining tooling.

Both pure machining and the WAAM tooling are part of the INNOTOOL project [85] in the Clean Sky 2 program within the Horizon 2020 framework, whose objective is the development of a thermoplastic press-forming tool for an advanced rear-end closing frame prototype and tooling 4.0 for the assembly and transportation of the advanced rear-end prototype. This rear-end prototype consisted of two symmetrical thermoplastic parts, each created using a symmetric mould, whose core part was created following the two stated manufacturing procedures. These similar toolings (Figure 4) could be assimilated as equal, allowing for a direct comparison between their energy and material consumption inputs. Both toolings achieve final dimensions of 500 × 450 × 150 mm.

The final products obtained by the two twin toolings were two high-load composite frames, which were integrated into an aircraft rear-end fuselage demonstrator. Composite materials respond to the current trend of reducing metallic materials in the transport industry [86,87]. This rear-end demonstrator was built up for validation purposes regarding safety certification and to structure the systems’ integration concepts up to full scale.

The process performed using these toolings was a press-forming procedure, in which a thermoplastic sheet is heated to a high temperature and fed into the tooling (core and cavity) and where, through a high-speed procedure, high pressure and high temperature are applied to produce a final part. This high temperature in the tooling is achieved by internal channels, through which high-temperature oil circulates. To achieve uniformity in the heating of the mould, the design attempts to adapt the oil channels to the geometry of the final part in the most appropriate way [88,89]. Also, a minimum weight is sought in the tooling so that its thermal inertia decreases, and, therefore, its cycle times. The tooling design was adapted to each manufacturing process, as described below. Also, at this point, both processes are described.

#### 2.1.1. Tooling Differences

**Figure 5:**
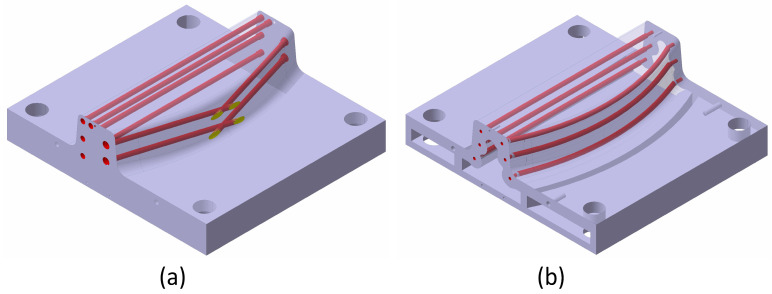
CAD design of both tooling, with internal channels highlighted in red (**a**) Pure machining tooling CAD design and (**b**) WAAM tooling CAD design.

The design differences between AM and pure machining toolings are highlighted in this section. Each process has its own singularities and restrictions that apply to each CAD design. Figure 5 displays the WAAM tooling’s final design and pure machining tooling’s final design. In both of them, their inner channels are highlighted. This figure also displays the hole plugs in the mould area of pure machining tooling. These differences are outlined and justified below.

Pure machining tooling:

1. Only a final design CAD is needed.
2. The internal channels can only be designed straight due to machining limitations. For this reason, they cannot be adapted to the final shape of the tooling. These drills are designed straight through the tooling and drilled in a step before finishing.
3. In an extra process, these drills are subsequently plugged (manually) and re-machined, as shown in Figure 5.
4. All construction singularities (threaded holes, reference holes, and holes for guideways) are performed during the same procedure.
5. Due to machining area limitations, the inner tooling part is not emptied, as in WAAM tooling.

**Figure 6:**
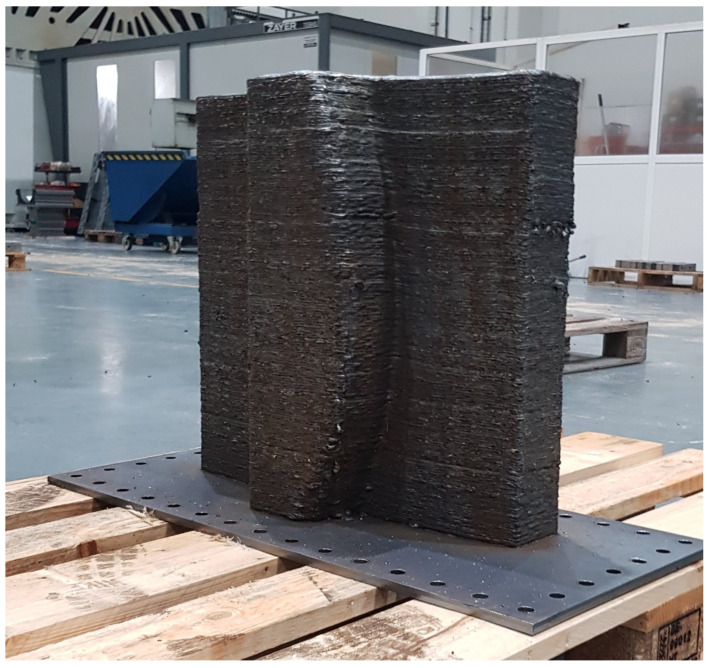
WAAM tooling printing over a metallic plate.

WAAM Tooling:

1. Any construction singularity, such as screw drills located on surfaces perpendicular to the growth direction, is eliminated during the WAAM design and machined in the posterior hybrid-machining process.
2. The main tooling is constructed over a metallic plate larger than the tooling used as a printing base. When the WAAM process starts, extra layers are constructed over the plate and eliminated in the hybrid machining procedure, as shown in Figure 6.
3. This figure also displays the printing direction, which is perpendicular to the theoretical base of the mould. This arrangement allows us to cast the inside of the part.
4. As highlighted in Figure 5, the final tooling has internal heating channels. These channels follow the curved surface of the final thermoplastic part, homogenising the temperature distribution in the mould and increasing its heating rate.
5. The design respects the restriction of a growth angle smaller than 20° relative to its horizontal plane.
6. The printing process allows the tooling inner part to be designed as empty (design supported by FEM analysis).

#### 2.1.2. Pure Machining Tooling

The pure machining process covers all roughing, drilling, and finishing processes from a raw steel block of nominal dimensions plus 5 millimetres of growth. These shape specifications cause the initial block size to be 510 × 460 × 158 mm. First, the bores used for positioning the part in the machine were drilled. Then, the drill holes for the thermal channels were produced. After that, a rough procedure eliminated any existing extra material. Then, drilling operations were conducted, and the finishing process was used to reach dimensional tolerances where needed.

As mentioned, the inner heating channels need to be manufactured in three stages:

1. First, a drilling process was carried out before the roughing process, creating through-holes that pass through the workpiece.
2. Then, rough milling of the area was carried out, approximating its geometry to the final geometry.
3. Afterwards, a re-tapping procedure was conducted to blind these through holes, creating the internal heating circuit.

**Figure 7:**
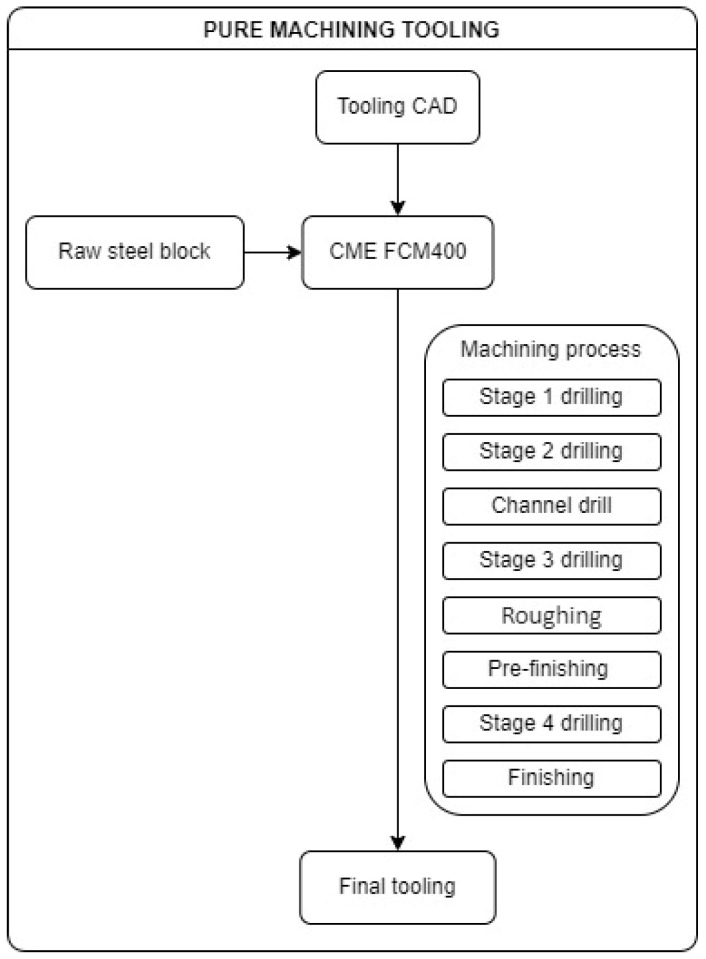
Pure machining tooling manufacturing diagram.

Finally, leftover material from the plugs used in the channels was removed in the subsequent pre-finishing and finishing procedures. The following diagram (Figure 7) shows an overview of the machining processes carried out on the NC machine.

#### 2.1.3. WAAM Tooling

First, the input material for the AM process must be selected. For this BAAM process, the chosen wire material was G42 4 M21 3Si1 (ISO specification). This carbon steel electrode offers excellent weldability, with many deoxidising elements for welding in which strict cleaning practices cannot be followed.

**Figure 8:**
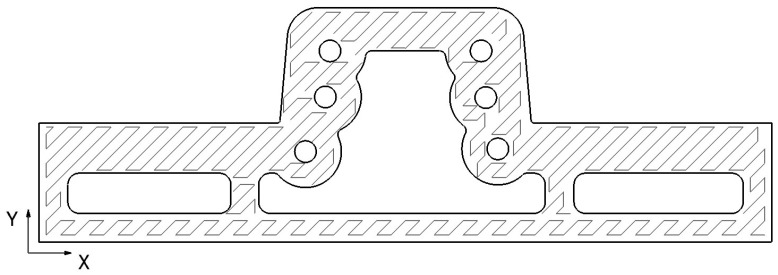
WAAM process printing route details. The lines shown represent the route followed by the WAAM system to build each layer, with the darker lines indicating the contours of the workpiece.

The printing strategy carried out for the WAAM process was the following:

1. Over the aforementioned metallic plate, five extra layers of the inferior final figure were printed. This strategy assures the orthogonality of the final construction and creates an extra height that ensures the ease of plate removal.
2. Based on the Figure 8 cutting, the WAAM strategy was the following for each layer:
  - First, the contour of the tooling was printed.
  - In the second stage, the contour of the inner channels was printed.
  - Subsequently, the inner part was fulfilled following Figure 8’s pattern, ending the layer.
  - The next layer started on a randomly selected point of the contour, avoiding, in this way, heat concentrations in specific areas.
3. The WAAM process input data are provided in Table 1.
4. The WFR is variable and adaptable to the geometry, printing route, and weld specifications. Its main value is also shown in Table 1.

Once the AM process ends, WAAM tooling hybrid machining starts with a cutting procedure to separate the printing base plate from the final tooling. After that, the roughing process only mills the areas that are to be subsequently subjected to drilling or finishing processes and the areas that require the removal of extra material printed in the WAAM process due to mechanical or procedure specifications.

The internal channels of the WAAM tooling were designed based on the experience gained from previous constructions using the WAAM method, as well as from previous tests. Their design and manufacture are considered successful after testing the internal channel system with thermal oil.

**Figure 9:**
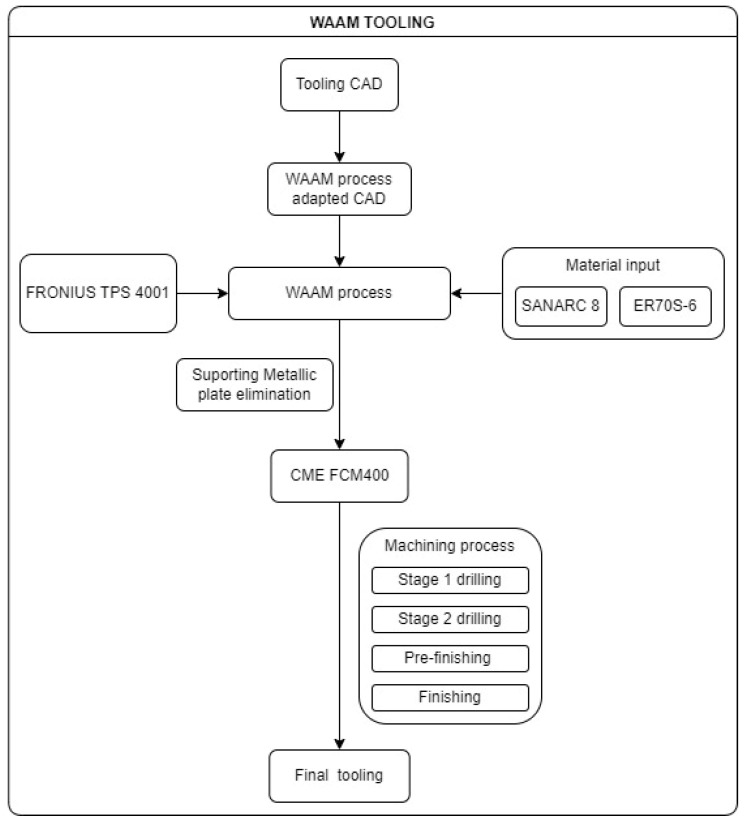
WAAM tooling manufacturing diagram.

The following diagram (Figure 9) summarises the tooling manufacturing, including an enumeration of the different machining processes carried out on the NC machine.

## 3. Results

### 3.1. Pure Machining Tooling

#### 3.1.1. Energy Consumption

Energetic input information for this procedure was characterised by the measured inputs obtained in real-time. In the case of pure machining tooling, the whole process was performed by the CME FCM400 and lasted 9.69 h. This procedure includes the drilling and rough milling channels and a pre-finishing and finishing step. The manual channel plugging step time was not included. The total amount of electric energy consumed across the process was 696.228 kWh.

#### 3.1.2. Material Consumption

**Figure 10:**
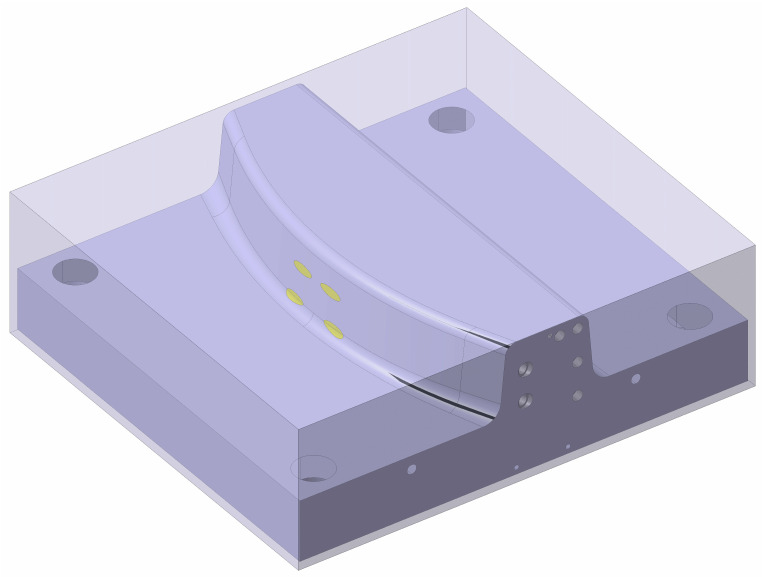
Raw material vs. final tooling in pure machining tooling.

For the pure machining tooling, as aforementioned in the Milling Process section, the original raw material was a 510 × 460 × 158 mm raw steel block (Figure 10), whose density was 7860 kg/m^3^. Two stages in Table 2 related to tooling weight are differentiated:

1. The material consumed weight.
2. The final tooling weight.

**Table 2:** Pure machining tooling material consumption.

Pure Machining Tooling	
Material consumed weight	291.345 kg
Final tooling weight	152.747 kg
Material discarded	47.57%

These states refer to the amount of raw material consumed in the process (raw steel block in this case) and, secondly, the mass of material held in the final tooling. From these two states, it is possible to find the percentage of the material used concerning the material expended and, therefore, the amount of material discarded in each procedure.

### 3.2. WAAM Tooling

#### 3.2.1. Energy Consumption

The technology required to perform the WAAM process, based on Kraken products, requires specific equipment to allow the welding system to work. All the elements around the AM system are already summarised in Table 1. Regarding support systems around the weld, all the energetic inputs are reviewed in Table 3. Under the concept of ‘General’, all installed sensors and control software are summarised. It must be mentioned that the Kraken MAM system only actuates the gantry crane system when the construction size exceeds the operating range of the robotic arm. In this case, the final construction dimensions are achievable by the robotic arm, so gantry crane fuel inputs are considered negligible. These consumptions were previously characterised during actual WAAM operations before the study.

The global WAAM printing process lasted 37.48 h in terms of the pure printing time. Throughout that time, the printing software recorded the position, wire feed rate, electric current, arc voltage, and layer height data. It must also be specified that, during additive manufacturing, no extra stop was required for cooling the workpiece. This stabilisation stop is common in WAAM systems. However, due to the large size of the part, the printing time itself was long enough to stabilise the part temperature.

It should be highlighted that the recorded data were post-processed, eliminating welding stops. This decision allows us to focus on the power consumption data obtained during the AM process, facilitating its later treatment. However, these times added to the welding were recorded and used for the final calculation of the power consumption and process times. The downtime measured during the process amounted to 4.58 h. These stoppages were due to the following causes:

1. The programming robot travelling between welding cordons.
2. The programming robot travelling between layers.
3. Not programming stops due to welding material replacement (wire or gas).

**Figure A1:**
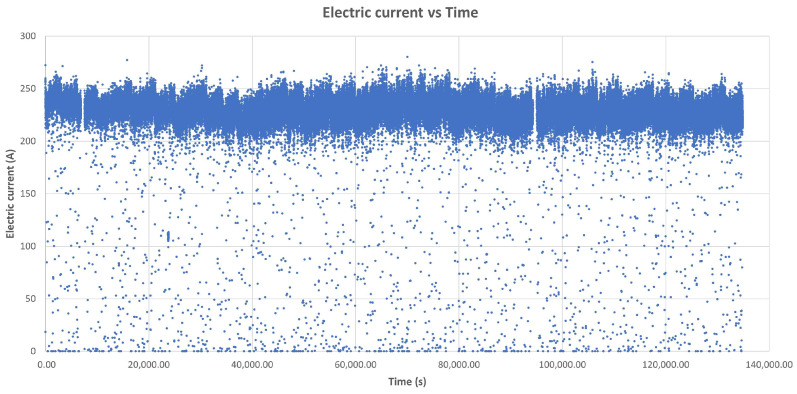
Electric current over time. The blue dots represent electric current data collected every 0.1 s throughout the entire WAAM manufacturing process.

**Figure A2:**
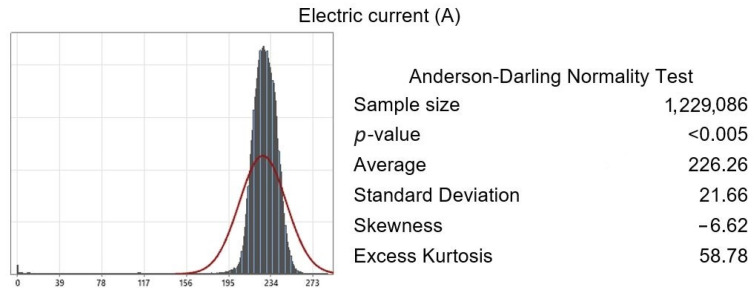
Anderson–Darling normality test applied to electric current data collected every 0.1 s throughout the WAAM manufacturing process. The *p*-value < 0.05 shows that the acquired data follow a normal distribution.

**Figure A3:**
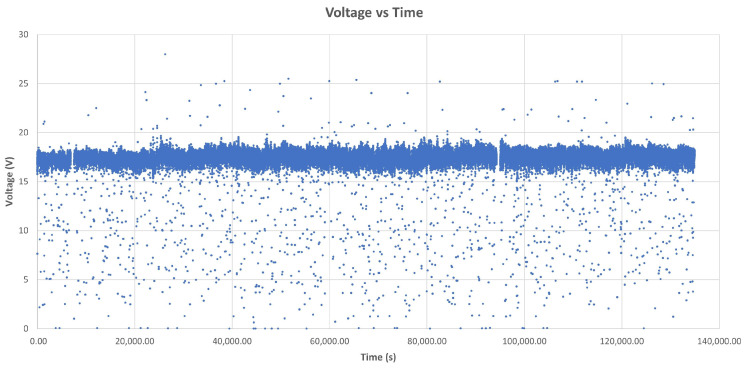
Voltage over time. The blue dots represent voltage data collected every 0.1 s throughout the entire WAAM manufacturing process.

**Figure A4:**
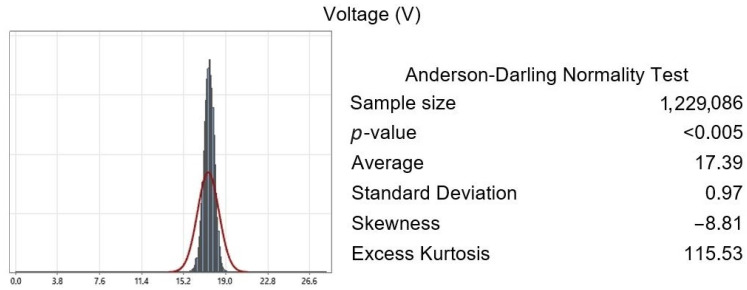
Anderson–Darling normality test applied to voltage data collected every 0.1 s throughout the WAAM manufacturing process. The *p*-value < 0.05 shows that the acquired data follow a normal distribution.

**Figure A5:**
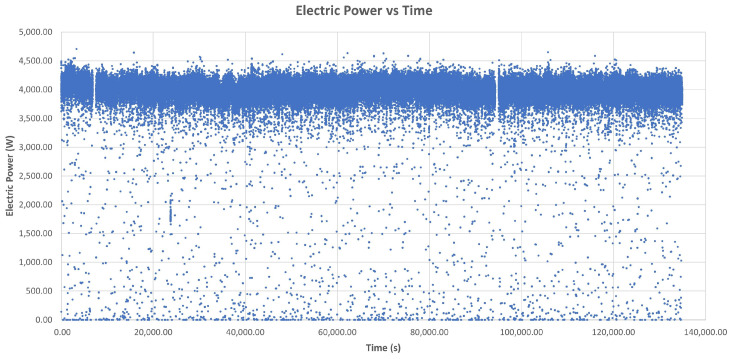
Electric power over time. The blue dots represent electric power data collected every 0.1 s throughout the entire WAAM manufacturing process.

**Figure A6:**
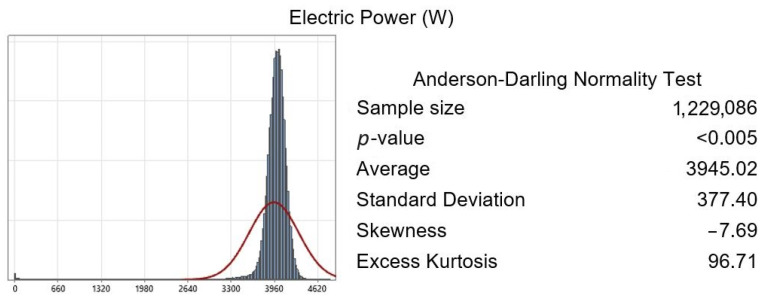
Anderson–Darling normality test applied to electric power data collected every 0.1 s throughout the WAAM manufacturing process. The *p*-value < 0.05 shows that the acquired data follows a normal distribution.

This article focuses on electric consumption, dependent on the electric current (Figure A1 and Figure A2) and arc voltage (Figure A3 and Figure A4). Both inputs follow a normal distribution; consequently, Figure A5 and Figure A6 also comply with this distribution. The large number of atypical values recorded in graphical areas between 0 and the lower statistical variance value should be mentioned. These atypical values could result from the WAAM process stability across the weld, which could be affected by different external inputs. These inputs, in general terms, result from robotic arm positioning errors or process singularities, such as specific areas with a large grouping of printing points, layer changes, or alternation between welding seams.

The total amount of electrical energy consumed by the system, combining the consumption of the WAAM process itself, the consumption of the robotic arm, and the control software, is shown in Table 4.

Once the AM process ends, the same NC machine is used to manufacture the pure machining tooling; therefore, the machining system inputs are the same as those presented in Table 5. The machining process energy consumption (311.365 kWh) overcomes the WAAM process (245.259 kWh). However, the sum of the two (568.515 kWh) is still less than the single tooling machining process of pure machining tooling (696.228 kWh). Under the concept of WAAM process energy consumption, we include both the pure consumption of the AM process (245.259 kWh) and the consumption of the system during secondary operations (11.891 kWh).

Referred to as WAAM tooling, the primary printing procedure eliminates the channel drilling and roughing processes (the most time-consuming) and keeps only the pre-finishing and finishing processes. Thanks to this step reduction, the machining time falls to 3.66 h.

#### 3.2.2. Material Consumption

**Figure 11:**
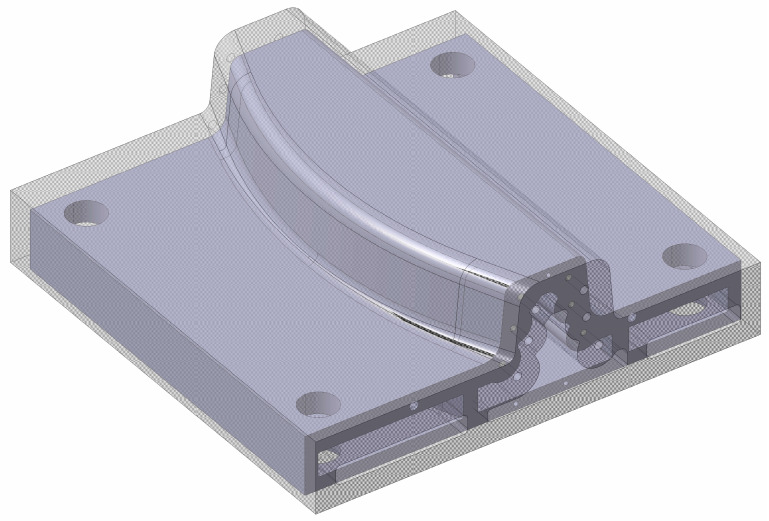
Raw material vs. final tooling in WAAM tooling.

**Figure A7:**
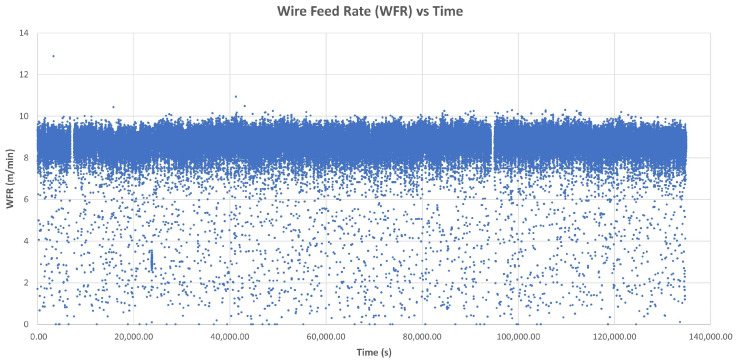
Wire feed rate over time. The blue dots represent WFR data collected every 0.1 s throughout the entire WAAM manufacturing process.

**Figure A8:**
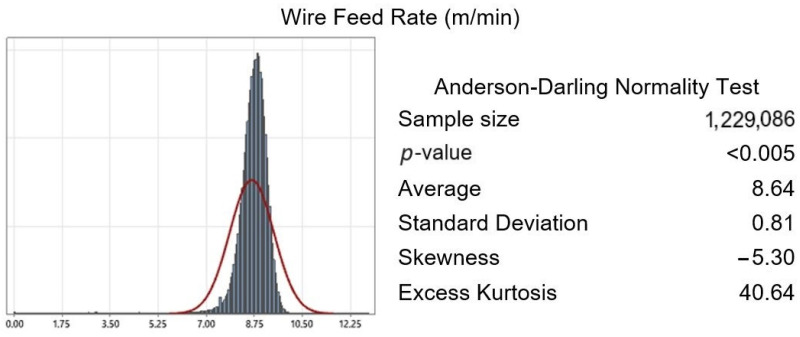
Anderson–Darling normality test applied to WFR data collected every 0.1 s throughout the WAAM manufacturing process. The *p*-value < 0.05 shows that the acquired data follow a normal distribution.

Regarding material consumption, specific considerations mentioned in Section 2.1.3 were considered. Figure 11 shows the WAAM tooling CAD design versus WAAM-process-adapted CAD. The recorded data of WFR determined the WAAM process material consumption. The statistical analysis of the data collected from the WFR is presented in Figure A7 and Figure A8, which follow a normal distribution. The total wire expenditure weight is shown in Table 6. 

The final results of material consumption and material discarded for WAAM tooling are shown in Table 6.

### 3.3. Sensitivity Analysis of the WAAM Over-Thickness

Once the data obtained from the WAAM tooling are processed, the question arises as to whether the additive manufacturing process could be optimised in such a way that it would be even more favourable in terms of electricity and material consumption. As previously mentioned, over-thickness is a safety measure considered in the design phase of the parts manufactured using WAAM technology, whereby a part thickness more significant than the final thickness is designed to minimise the effect of defects in the welding procedure. This over-thickness is machined later in the precision-finishing phase in the necessary areas. Based on this premise, several over-thicknesses were tested theoretically for the WAAM-process-adapted CAD on which the tooling AM process is based. It should be mentioned that the 10 mm over-thickness given to the original CAD responds to conservative criteria, seeking to minimise the possibility of errors and not the optimisation of the procedure.

To carry out this study, the WAAM-process-adapted CAD was modified, increasing the over-thicknesses to a final 20 mm and reducing it to 1.5 mm. Considering that the WAAM-process-adapted CAD cannot have an over-thickness of 0 mm, as the very concept of over-printing layers in the WAAM process would make it impossible not to incur areas of material shortage, a minimum over-thickness of 1.5 mm was used to optimise the print as much as possible while trying to avoid such areas of material shortage. On the other hand, logic dictates that any design thickness greater than the original 10 mm would increase both material consumption and energy costs. However, higher values of over-thickness will be tested to examine the implications of consumption. For these over-thicknesses of more than 10 mm, the advantages derived from the AM process were reduced or even disappeared. The WAAM-process-adapted CAD derived from high over-thicknesses makes it impossible to perform the internal emptying of the part. Consequently, the tooling cannot be made lighter than the pure machining tooling. However, values of less than 10 mm significantly reduced material consumption.

#### 3.3.1. Theoretical Energy Input

**Figure 12:**
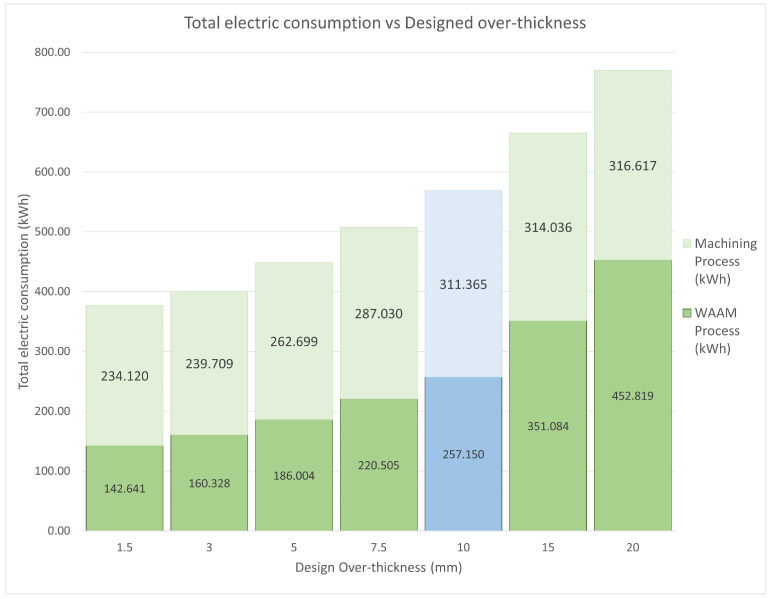
Theoretical over-thickness energy inputs. Green columns display theoretical results meanwhile blue column shows the actual data obtained.

The theoretical results obtained from the performed toolings are shown in Figure 12. This graph shows the energy expenditure for the additive manufacturing procedure and the subsequent second-stage hybrid-machining process.

The WAAM tooling is not machined in its casting areas in any case, and the function of these zones is only weight reduction; therefore, they do not require any machining process. These areas only represent the additive manufacturing phase, and only the functional part of the tooling shall be machined.

It is also worth noting that when the machining thickness exceeds 10 mm, an extra machining process, rough milling, should be added to the ones already applied to the WAAM tooling. Nevertheless, rough milling is much less energy-consuming than the later finishing operations, so its relevance to the total energy consumption is minor.

The calculated energy inputs show a higher relevance of the over-thickness parameter in the WAAM printing process than in the subsequent machining. In the case of the most negligible printing thickness, 1.5 mm, energy savings of 44.53% are achieved in the WAAM process and 24.81% in the subsequent machining, with a total theoretical energy saving of 33.73% for the original WAAM tooling manufactured. On the other hand, for the most unfavourable case of over-thickness, 20 mm, the terms of energy consumption soar. The power consumption of WAAM printing increases by 276.09%, and that of the subsequent machining increases by 201.69%, compared to the original 10 mm WAAM-process-adapted CAD. Consequently, the total electric consumption increases by 235.34%. As mentioned above, the most electrically demanding machining procedures are the finishing processes, which are the same for all toolings 10 mm thick or more. From this thickness, a rough milling process was carried out until this thickness was reached, making this procedure much less electrically demanding. For this reason, the machining energy consumption does not increase linearly with the WAAM print consumption from 10 mm over-thickness.

#### 3.3.2. Theoretical Energy Input

**Figure 13:**
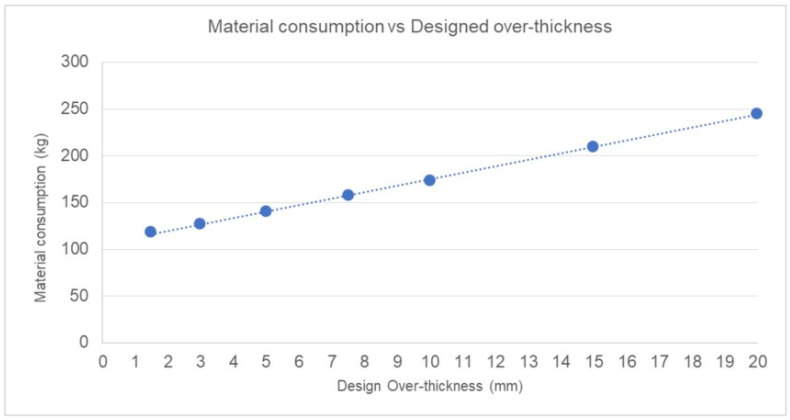
Material consumption vs. design over-thickness. The graph shows the linear growth of the amount of material consumed during the WAAM process (kg) with reference to the over-thickness considered in WAAM-process-adapted CAD.

As in the previous section, the theoretical result of material consumption during AM operations, based on the WAAM tooling results, is shown in Figure 13.

The minimum 1.5 mm over-thickness of the WAAM-process-adapted CAD over-thickness displays a theoretical reduction of 32.01% of the raw material consumption compared to the 10 mm thick construction. This results in a better use of material, reducing the percentage of theoretically discarded material to 22.70% of the final tooling.

On the other hand, for over-thicknesses greater than 10 mm, the increase in material theoretically consumed scales up to 241.90% in the worst case (20 mm over-thickness). Consequently, the discarded material increases, although in several areas, this material is not removed in the second-stage hybrid machining, increasing the final weight of the tooling. This would be the case of the aforementioned inner voids, which, due to this over-thickness, would practically disappear and later challenge the machine.

**Figure 14:**
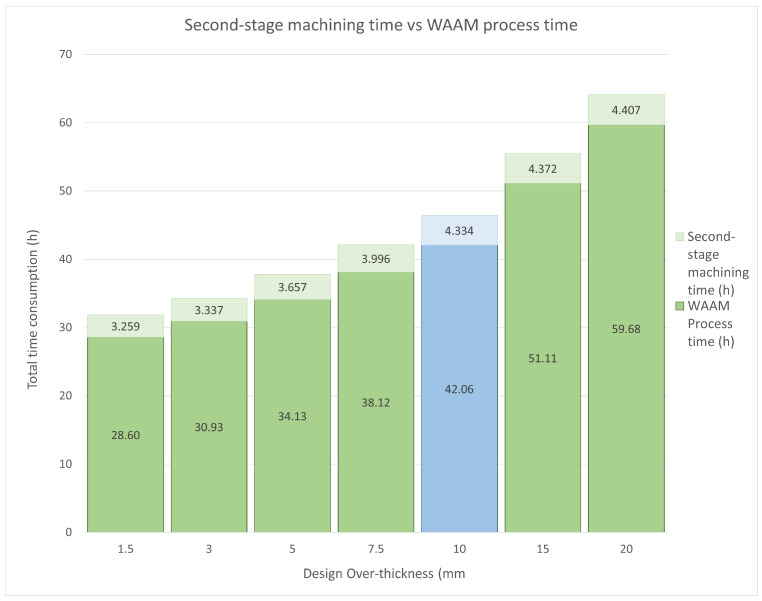
Hybrid machining time vs. WAAM process time. Green columns display theoretical results meanwhile blue column shows the actual data obtained.

Finally, the last parameter to be commented on is the process time. Figure 14 displays the total consumed time with the accumulation of the WAAM process time plus the hybrid machining time. As observed, the AM process time covers most of the entire process time. decreasing by 32.01% for the most favourable case compared to a manufactured tooling over-thickness of 10 mm. For the hybrid machining process times, as mentioned above, by increasing the over-thickness to more than 10 mm, a rough milling procedure was added to the operations to be carried out. This procedure is faster than the finishing milling processes, so the increase in time was less significant.

As shown in Figure 14, the most favourable total process time with a 1.5 mm over-thickness is 31.855 h, and the most unfavourable time at a 20 mm over-thickness is 64.092 h. Even in the most favourable scenario, the WAAM tooling manufacturing process continues to be three times slower than pure machining manufacturing.

#### 3.3.3. WAAM Tooling Metrology

After the AM operation, a metrology studio was performed against the WAAM tooling. Using a T-SCAN type measurement scanner [83], a 3D point cloud was obtained. This point cloud was compared with the WAAM-process-adapted CAD through a best-fit procedure, and the metrology studio was performed against the WAAM-process-adapted CAD.

**Figure 15:**
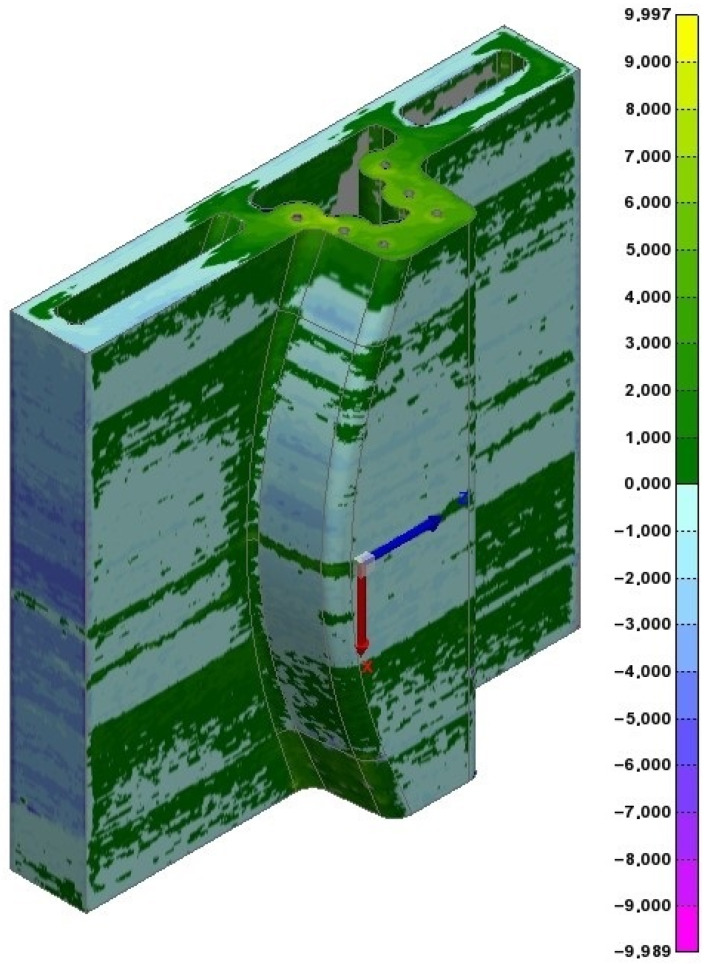
WAAM tooling metrology studio. Data are shown in mm.

The results clearly show differentiated zones in which over-thickness layers are interspersed with large areas of below-grade thicknesses (Figure 15). This zone differentiation clearly shows the construction boundary layer between the sections, with excess material compared to the theoretical value and those with a lack of material. This effect is caused by the thermal expansion of the material during the additive process. As explained previously, the WAAM process deposits material at high temperatures and is thus in a state of thermal expansion. This characteristic is considered in the design process. As a result, the final part shrinks slightly when the process is finished and cools down. If the entire part is printed continuously, the thermal shrinkage affects the entire workpiece. However, if a prolonged stop is made throughout the build process, the workpiece shrinks and shifts its geometry slightly away from the printing route. Therefore, when the printing process is resumed at the same point, the printed layer is displaced in the direction of shrinkage, causing the next layer not to be deposited fully aligned with the previous one. Accordingly, this new weld bead will not be deposited in its ideal position concerning the previous layer but deposited off-centre, creating an overhang and, therefore, increasing in width and decreasing in height.

For this reason, the areas in which construction stops tend to be out of level, having excessive thicknesses. This feature is not critical for tool printing and subsequent second-stage hybrid machining, as these wider layers will be machined afterwards in any case. This anomaly is clearly visible in the image, with weld beads deposited after a process stop.

One important point to highlight is that the extra deposited material is not problematic for ensuring the quality of the final product, and areas with excess material are mechanised in subsequent procedures. However, under-material areas can be problematic if the under-material exceeds the over-thickness designed in the WAAM-process-adapted CAD, as it makes it impossible for the final part to reach the dimensions after the second-stage hybrid-machining process.

**Figure 16:**
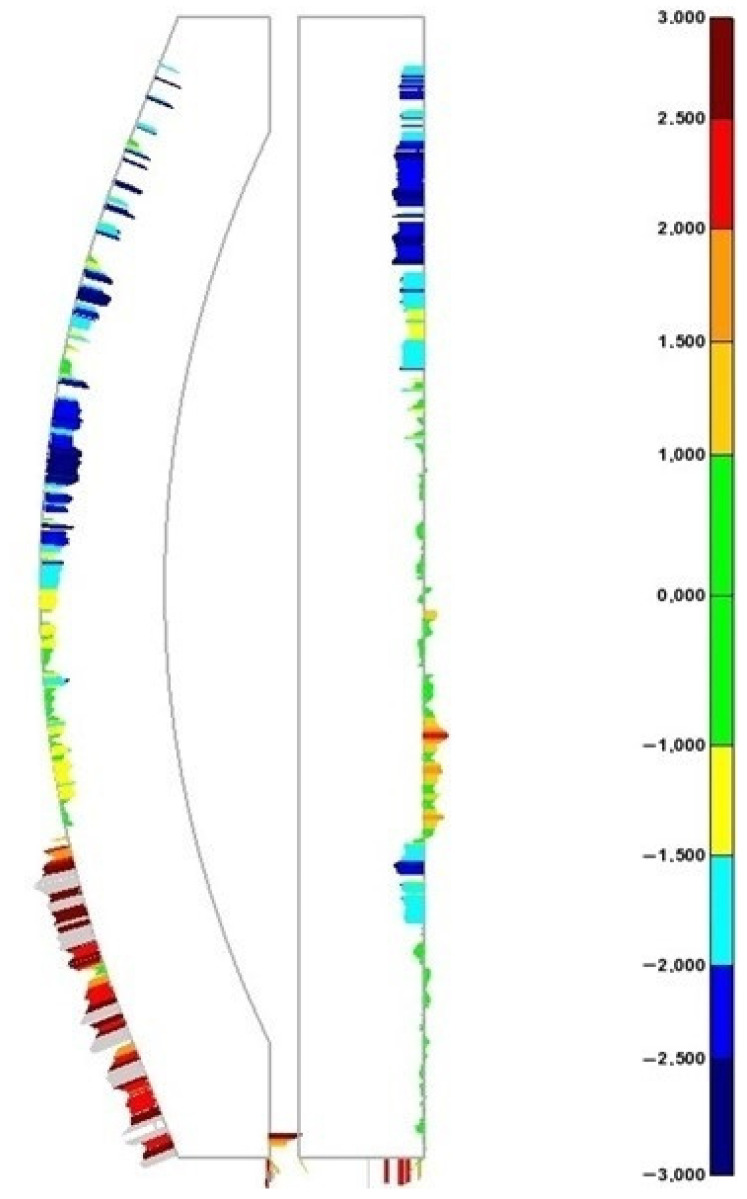
WAAM tooling metrology studio—punch section. Data are shown in mm.

During the metrology process, several studios were made of the section of the punch area of the mould, as this was the most compromised tooling area. Of the multiple measurements performed, the most unfavourable one was taken, as shown in Figure 16. In this image, the mentioned thermal dilatation process could be appreciated.

The data obtained from the metrology studio show a −3 mm maximum negative AM deviation concerning the WAAM-process-adapted CAD in the areas where the lack of material is more accentuated. These data suggest that a minimum 3 mm over-thickness should be applied to the WAAM-process-adapted CAD in order to obtain the first AM product available to achieve the final tooling geometrical specifications. This fact discards the calculated theoretical over-thickness of 1.5 mm but makes the next estimated value of 3 mm feasible. The values of the positive deviations overcome the 3 mm in the tested area, although these positive values do not represent a constructive issue, as extra material is later machined.

## 4. Discussion

In conclusion, the results showed a reduction in material consumption and electric inputs for the WAAM tooling compared to the pure machining one.

As mentioned, WAAM process advantages are oriented to design and construct tailored and complex geometries, which is impossible to achieve by conventional manufacturing methods. Nevertheless, this study displays the capacity of this AM procedure to save energy and material in specific constructions.

The obtained results for this particular tooling design showed a total energy consumption of 696.228 kWh for pure machining tooling. On the other hand, the recorded data showed a combined energy consumption of 568.515 kWh for WAAM tooling, combining both WAAM and hybrid machining. Based on the data obtained, a significant reduction in energy consumption (18%) was achieved in the WAAM tooling for pure machining tooling.

It must be highlighted that, in a high percentage, the weight difference between the final tools developed by the two differentiated manufacturing processes is primarily due to their design differences. These differences are the consequences of the specifications of each manufacturing process, as named in Section 3.1.1, Tooling Differences. The adequacy of the procedure is closely related to the complexity of the final design. In cases such as the current design, its complexity lies in the ability to decrease its mass, thereby reducing its thermal inertia. Since it is a press-forming tooling, it is desirable to minimise the thermal cycles.

In this specific design, the internal hollows of the WAAM tooling significantly reduce its weight concerning the pure machining tooling. In this case, WAAM-produced tooling weight represents a 41% reduction regarding the pure machining tooling. This final weight reduction could be defined as a design-dependent property, the percentage of which can vary widely depending on the final design.

On the other hand, the material discarded shows the relation between the raw material input and the final material used, displaying an almost equal material utilisation percentage in both toolings. Nevertheless, the difference in the final weight between the two toolings means that, although the percentage of material utilisation is very similar, the amount of discarded material is much higher in pure machining tooling.

The difference in the construction time of the tooling should be discussed. In the case of pure machining tooling, the entire procedure is carried out on the same NC machine. This represents a significant time saving compared to the WAAM process, where the manufacturing has two steps: an AM process and a machining process. In addition, the transport of the raw tooling printed by WAAM to the NC machine should be mentioned; nevertheless, in this case, it has been omitted as it is of negligible value.

With all these specifications, we obtained a total manufacturing time for the pure machining tooling of 9.7 h for the whole procedure. In contrast, for the WAAM tooling, we first had an AM process that lasted 42.07 h (combining 37.48 h of the pure WAAM process with 4.58 h of downtime) followed by a machining process of 4.3 h, adding up to a total of 41.8 h.

The results also conclude that the WAAM process is four times more time-consuming than pure machining for this tooling design. This characteristic of AM processes makes their use in BAAM processes unfeasible when large batches of parts are to be created in short periods of time.

Based on the theoretical analysis performed for different design over-thicknesses for the WAAM-process-adapted CAD, there is an opportunity for a further reduction in energy and material costs. Once the metrological analysis was concluded, the results supported that a decrease in the over-thickness from 10 mm to 3 mm for the WAAM-process-adapted CAD would be optimal. The reduction in the design over-thickness linearly decreases both parameters, as well as the manufacturing time, achieving savings of 43.8% in total energy expenditure and 56.3% in raw material compared to the pure machining tooling. On the other hand, although the total manufacturing time of the theoretical WAAM tooling also following a decreasing trend with the reduction in design over-thicknesses, this most favourable case still requires a total manufacturing time three times longer than pure machining tooling.

It should also be noted that this study is focused on the manufacturing processes, thus limiting this particular case to a gate-to-gate study. Future lines of research in this field should focus on the complete analysis of the whole life cycle of the manufactured part, conducting a cradle-to-grave study.

## 5. Conclusions

In summary and conclusion, the suitability of the WAAM manufacturing process for producing large-sized tooling was demonstrated. For the specific case analysed, the reduction in electrical and material consumption was 18% and 41%, respectively, compared with pure machining tooling. This weight reduction also minimised the thermal inertia in the process, increasing its productivity. Although this study was gate-to-gate, focusing on the energy needed for the additive and subtractive manufacturing process of the tooling, it did not consider the energy needed to produce the raw block, welding wire, and shielding gas.

On the other hand, the manufacturing time employed in WAAM tooling is higher than that required by pure machining tooling. However, since WAAM tooling printing is a continuous process, it only requires surface-finishing operations, being less time-consuming. In contrast, pure machining tooling requires complex positioning processes to perform challenging operations, such as thermal channels. These operations can significantly increase the manufacturing time, minimising the difference in the manufacturing time.

In addition, the metrological analysis of the WAAM tooling revealed an optimisation capacity at the design and manufacturing level. In the most favourable case, a theoretical reduction in energy and material consumption of 44% and 56%, respectively, could be obtained compared with the pure machining tooling. This theoretical approach will be studied in future constructions.

In this study, a gate-to-gate manufacturing process was analysed for two aeronautical toolings designed to manufacture limited batches. The study of the durability of both tooling and its comparison was not contemplated due to their reduced productivity. This WAAM tooling durability is considered a possible line of future research, in relating this characteristic to the mechanical properties of the WAAM tooling. In this context, an interesting line of research is the analysis of the life cycle of the WAAM tooling, not limited to its production phase, but to a cradle-to-grave analysis.

As discussed throughout the article, the data obtained depend on the final part design. However, the procedures mentioned are common for tools manufactured by additive manufacturing procedures, so although it is not possible to create a rule regarding the proportionality between manufacturing times, it serves as a guideline.

## Figures and Tables

**Table 1:** WAAM process parameters.

Process Parameters for WAAM Deposition		
Process parameters	Details	Value
Speed	Welding speed	0.02 m/s
	Wire feed rate	8.64 ± 0.81 m/min
	Deposition rate	0.077 kg/min
Distance and angle	Layer height	1.2 mm
	Electrode to layer angle	90°
Shield gas	Shield gas type	ISO 14175-M20-ArC-8 [90] (CO_2_ 8% Ar 92%)
	Shield gas flow rate	15 L/min

**Table 3:** KRAKEN system power consumption.

Energy Inputs	Average Power Consumption
Gantry crane	1.890 kWh
Robotic arm	0.440 kWh
General	2.15 kWh

**Table 4:** WAAM process deposition power.

WAAM Process Deposition Power	
Current	226.26 ± 21.66 A
Arc voltage	17.39 ± 0.97 V
Electric power	3945.00 ± 377.36 W

**Table 5:** Total electric energy consumption during WAAM tooling.

	Machining Process (kWh)	WAAM Process (kWh)	TOTAL (kWh)
WAAM Tooling	311.365	257.150	568.515

**Table 6:** WAAM tooling material consumption.

WAAM Process Deposition Power	
Material consumed weight	173.091 kg
Final tooling weight	90.960 kg
Material discarded	47.45%

## Data Availability

The data supporting the findings of this study are available from the corresponding author upon reasonable request.
